# Arrhythmic Risk Stratification in Cardiac Amyloidosis: A Review of the Current Literature

**DOI:** 10.3390/jcdd11070222

**Published:** 2024-07-14

**Authors:** Eleonora Bonvicini, Alberto Preda, Chiara Tognola, Raffaele Falco, Roberto Gidiucci, Giulio Leo, Sara Vargiu, Marisa Varrenti, Lorenzo Gigli, Matteo Baroni, Marco Carbonaro, Giulia Colombo, Alessandro Maloberti, Cristina Giannattasio, Patrizio Mazzone, Fabrizio Guarracini

**Affiliations:** 1 Department of Cardiology, S. Chiara Hospital, 38122 Trento, Italy; 2 Electrophysiology Unit, De Gasperis Cardio Center, Niguarda Hospital, 20162 Milan, Italysara.vargiu@ospedaleniguarda.it (S.V.);; 3 Clinical Cardiology Unit, De Gasperis Cardio Center, Niguarda Hospital, 20162 Milan, Italy; chiara.tognola@ospedaleniguarda.it (C.T.); alessandro.maloberti@ospedaleniguarda.it (A.M.);; 4 School of Medicine and Surgery, University of Milano-Bicocca, 20126 Milan, Italy

**Keywords:** cardiac amyloidosis, conduction system disorders, risk stratification, heart failure, sudden cardiac death

## Abstract

Cardiac amyloidosis is the most frequent infiltrative disease caused by the deposition of misfolded proteins in the cardiac tissue, leading to heart failure, brady- and tachyarrhythmia and death. Conduction disorders, atrial fibrillation (AF) and ventricular arrhythmia (VA) significantly impact patient outcomes and demand recognition. However, several issues remain unresolved regarding early diagnosis and optimal management. Extreme bradycardia is the most common cause of arrhythmic death, while fast and sustained VAs can be found even in the early phases of the disease. Risk stratification and the prevention of sudden cardiac death are therefore to be considered in these patients, although the time for defibrillator implantation is still a subject of debate. Moreover, atrial impairment due to amyloid fibrils is associated with an increased risk of AF resistant to antiarrhythmic therapy, as well as recurrent thromboembolic events despite adequate anticoagulation. In the last few years, the aging of the population and progressive improvements in imaging methods have led to increases in the diagnosis of cardiac amyloidosis. Novel therapies have been developed to improve patients’ functional status, quality of life and mortality, without data regarding their effect on arrhythmia prevention. In this review, we consider the latest evidence regarding the arrhythmic risk stratification of cardiac amyloidosis, as well as the available therapeutic strategies.

## 1. Introduction

Cardiac amyloidosis (CA) is the most frequent infiltrative cardiomyopathy worldwide [1]. In the last few years, its growing prevalence has been recorded due to the aging population, advances in diagnosis and therapeutic methods and finally the increased awareness of the medical population [2]. Indeed, from 2000 to 2012, there was a significant increase in prevalence (8 to 17 per 100,000 person-years) and incidence (18 to 55 per 100,000 person-years) [3,4].

**Figure 1:**
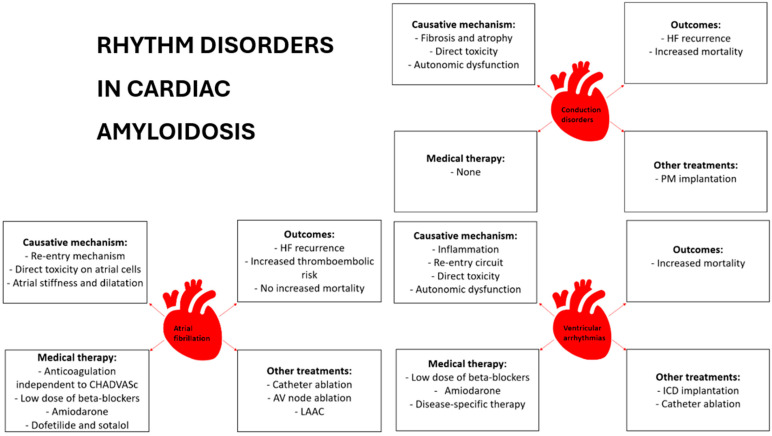
Etiopathogenesis, prognosis and treatment of arrythmia in cardiac amyloidosis.

Amyloidosis is caused by the deposition of misfolded and non-degradable proteins in the extracellular space of different tissue types; to date, more than 30 different proteins have been identified as responsible for amyloid formation due to genetic or acquired modifications, but only 10 proteins are known to be stored in the myocardium. Among them, immunoglobulins, light chains and transthyretin (TTR) [1] represent more than 98% of all cardiac amyloid precursors, with TTR being the most frequent type. The progressive accumulation of amyloids in the heart leads to hypertrophic remodeling and increased stiffness, resulting in diastolic dysfunction in the early stages [5]. Heart failure with preserved ejection fraction (HFpEF) is the earliest and most frequent presentation at the time of diagnosis [6,7]; systolic impairment follows, and it is characterized at the beginning by a reduction in left ventricle global longitudinal strain, associated traditionally with “apical sparing” [8,9]. Atrial wall dysfunction [10,11] and valve defects, like significant mitral regurgitation and aortic stenosis, due to direct amyloid deposition are also distinguishing features of CA [12]. Indeed, in patients over 65 years old, affected by severe aortic stenosis, cardiac amyloidosis is frequently encountered and needs to be excluded. Moreover, palpitations, syncope and sudden cardiac death (SCD) characterize the natural history of this disease, with atrial fibrillation (AF), ventricular arrhythmia (VA) and conduction disorders as the most frequently underlined findings. The arrhythmia prevalence is up to 60% according to studies [13], being associated with an increased rate of hospitalization and length of stay and a reduction in survival rates [14,15]. Table 1 and Figure 1 provide an overview of the prevalence, etiopathogenesis, prognosis and treatment of arrythmias in cardiac amyloidosis (CA).

It has to be considered that although amyloid deposition takes place in all human bodies, cardiovascular events represent more than two thirds of the casualties [16]; even if a portion of the cardiac deaths remain unexplained [17], arrythmias play a major role. The underlying etiology makes arrythmia management particularly challenging due to the labile hemodynamic stability and progressive nature of the disease. In this review, we provide a comprehensive overview of the arrhythmic management of patients with CA, suggesting tools for practical decision-making.

**Table 1:** Differences in rate of arrythmia in cardiac amyloidosis.

	Conduction Defects [18]	Atrial Fibrillation [19,20]	Ventricular Arrythmias [21,22]
AL	+	+	++
ATTRwt	++	++	+
ATTRv	+	+	+

## 2. Conduction System Disease

### 2.1. Epidemiology and Risk Stratification

Bradyarrhythmias are the most common and feared arrhythmic disorders in CA [23]. Several studies have reported electromechanical dissociation as the final stage of the disease, as well as the most frequent cause of death [24]. Amyloid fibrils’ infiltration of the conduction system, leading to fibrosis and the atrophy of the sinoatrial node, the atrio-ventricular (AV) node and the bundle branches, has been confirmed by autoptic findings [25,26]. Moreover, the direct toxicity of cardiomyocytes is the cause of apoptotic pathway activation as well as oxidative stress. Perivascular amyloid deposition and the subsequent impaired vasoreactivity and myocardial ischemia are also additional causes of electrical conduction impairment. ATTR leads to the slower progression of cardiac damage and higher quantities of fibers infiltrating the cardiac tissue compared to AL, which is characterized by a faster decline due to the greater toxic effect [27]. The greater prevalence of conduction defects has therefore been identified in ATTR patients [23]. Of note, as amyloid fibrils have an affinity for neurological cells [28], particularly AL and hereditary ATTR (ATTRv) [29,30], the involvement of the cardiac autonomic system, leading to vasovagal syncope and orthostatic hypotension, is a common concern in the natural history of CA. Yamada et al. [31] enrolled 55 AL patients with CA undergoing Holter monitoring and demonstrated a reduction in heart rate variability (HRV) and impaired heart rate turbulence (HRT), while Kastritis et al. [32] evaluated 50 AL patients through blood pressure (BP) monitoring and highlighted a decrease in blood pressure in patients with a poor prognosis. HRV, HRT and alterations in BP are parameters that have been considered to be vagally mediated and are therefore non-invasive markers of neurohormonal activity, and a reduction in the function of the autonomic nervous system has been linked to a poorer prognosis [32,33].

The differences in incidence between ATTR and AL in sinus node dysfunction and AV conduction system defects have been explored in retrospective cohorts, reporting 10% of high-grade AV blocks, 51% with a prolonged QRS complex and 49% with first-degree AV blocks in ATTR [34], with respect to the higher prevalence of intraventricular blocks in AL [35]. Overall, AV conduction defects prevail over sinus node disease [36,37], and a right bundle branch block is a mainstay even if the cause of the greater vulnerability of the right bundle is not certain; the smaller dimensions and greater exposure could impact this [38]. However, ECG alterations are usually manifested in advanced phases of conduction infiltration, as demonstrated by electrophysiological studies reporting the significant prolongation of the infra-His conduction time associated with moderate prolongations of the QRS. Reisinger et al. [37] identified an HV interval of 88 ± 17 ms and 77 ± 18 ms in AL patients with a prolonged and non-prolonged QRS, respectively. Similar results have been found in a more recent study conducted on 20 patients [39]. It is therefore speculated that only a severe injury of both branches causes ECG changes [37].

### 2.2. Anti-Bradycardia Pacing and Cardiac Resynchronization Therapy

**Figure 2:**
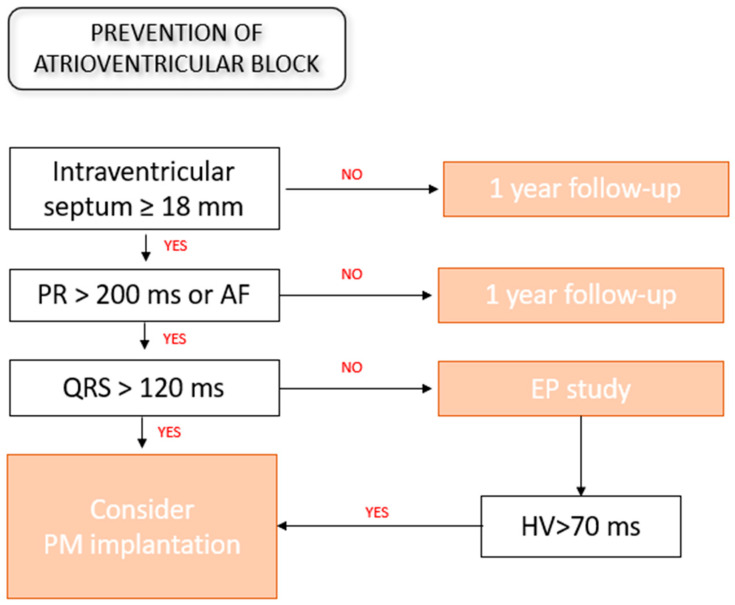
Decision tree for prophylactic PM implantation in cardiac amyloidosis. AF = atrial fibrillation, EP study = electrophysiological study, PM = pacemaker.

The preventive effect of pacemaker (PM) implantation on mortality has been a subject of discussion [18,40]. Pinney et al. demonstrated that, in ATTR wild-type (ATTRwt), a worse prognosis is correlated, alongside elevated troponin levels and class IV NYHA symptoms, with pacemaker implantation [41]; it is therefore suggested that conduction abnormalities requiring PM implantation are a sign of more advanced disease and an increased risk of sudden cardiac death. Another study on 20 carriers of implantable loop recorders (ILR) showed that a significant number of deaths were related to extreme bradycardia [42], supporting the indication of prophylactic PM implantation for the prevention of SCD. The progression of ATTRv is correlated with pacing dependency, as evidenced by follow-up records of patients undergoing PM implantation for syncope, ECG alterations and prolonged HV intervals [30]. Different records indicated a higher incidence of complete AV blocks compared to the general population [30,34]. Accordingly, another study demonstrated that the ventricular pacing burden increased from 35.5% at 6 months post-implantation to 96.2% at 5 years of follow-up [43]. In a meta-analysis on 735 patients undergoing transcatheter aortic valve implantation (TAVI), patients with CA had a 1.66-fold increased risk of PM implantation compared to controls [44]. Amyloidotic hearts are characterized by a fixed stroke volume and a heart-rate-dependent cardiac output. Since bradycardia is particularly detrimental in this setting, PM implantation to maintain a higher basal heart rate may lead to symptom relief and an improvement in quality of life. Nevertheless, no studies have been conducted to explore this hypothesis; therefore, no specific recommendations have been provided by guidelines [45,46]. Considering the natural history of the disease, a validated model to evaluate the risk of PM implantation is deemed necessary. Porcari et al. [18] conducted a study on 405 patients with AL and ATTR to identify predictors of PM implantation: a history of AF, PR > 200 ms and QRS > 120 ms resulted in an increased likelihood of the need for anti-bradycardia pacing. Similarly, a retrospective cohort study [40] of 778 patients reported the QRS duration and interventricular septum (IVS) thickness as independent predictors of PM implantation. Intriguingly, each millisecond increase in the QRS duration and each millimeter increase in the IVS thickness were related to a 2.6% and 10.6% increased risk of PM, respectively. These findings were partially confirmed by Dias de Frias et al. [47], who found, in vATTR patients, correlations with the neurological stage and the number of organs affected. In contrast, for NT-proBNP, the troponin value and left ventricle ejection fraction (LVEF) had no predictive power. In conclusion, ECG findings, including the PR and QRS duration, have been proven to be crucial for risk stratification. AF onset represents a turning point in the natural history of the disease [20]. The IVS thickness is correlated with early conduction disorders, as opposed to the LVEF, which changes only in the advanced stages. Electrophysiological (EP) studies to evaluate conduction delays are supposed to be helpful in uncertain cases [48]. In Figure 2, we provide a hypothetic decision tree for prophylactic PM implantation in CA. Finally, given the potential PM dependence and reduced hemodynamic reserves, prophylactic LV lead placement should be carefully considered due to the high probability of pacing-induced cardiomyopathy [49].

## 3. Atrial Fibrillation

### 3.1. Epidemiology and Risk Stratification

Atrial fibrillation (AF) is the most frequent tachyarrhythmia in CA, varying from 15% to 69% among studies [19,20,50] and overcoming by far the prevalence in the general population [51]. In particular, ATTRwt is more frequently linked to AF than the other subtypes, with prevalence > 40% [19,20,50,52]. Clinically, ischemic stroke can be the first presentation of atrial arrhythmia in CA patients, highlighting the importance of its early detection and management. Currently, the available studies show the significant impact of AF on cardiovascular compensation, leading to an increased risk of HF hospitalization without an influence on overall mortality [19,20,52,53,54]. AF development in CA is attributable—besides the increase in intra-atrial pressure due to diastolic dysfunction—to the deposition of amyloid fibers in the atrial and ventricular chambers with the subsequent disruption of the conduction system, leading to multiple re-entry mechanisms and the impairment of cardiomyocytes’ electrical properties, as macroscopically demonstrated by low atrial voltages on the surface ECG [55,56]. EP studies have shown extensive areas of left atrial low voltages with a greater number of inducible arrhythmias resistant to medical and interventional therapies [39]. Moreover, a number of additional risk factors linked to CA are well-known triggers of AF, as well as causing its persistence, including a reduced LVEF, increased filling pressures and a reduced estimated glomerular filtration rate [19,50]. In recent studies, atrial mechanical dysfunction showed a higher correlation than the atrial volume with AF development, underlining the toxic effect of amyloids on atrial cardiomyocytes [10,57,58]. It has also been demonstrated that increased atrial stiffness is responsible for primary atrial failure [10], predicting the AF incidence and early HF recurrence [59]. In a retrospective study, Lohrmann et al. [60] proposed the time to peak strain rate, a parameter for mechanical dispersion in the early reservoir phase of left atrial function, as a predictor of the development of AF in AL patients undergoing high-dose melphalan treatment and autologous stem-cell transplantation. Early studies included also autonomic dysfunction among the risk factors of AF [61]: in the Atherosclerosis Risk in Communities (ARIC) cohort, which enrolled 11,715 patients, it was reported that a higher incidence of AF was correlated with a low HRV.

### 3.2. Pharmacological Therapy

AF management in CA represents a major challenge due to the low effectiveness of the current treatments. Generally, atrial arrythmias are highly symptomatic and poorly tolerated due to rapid ventricular rates and irregular ventricular responses, which impair ventricular filling and contractility. Moreover, medications for rate control, such as beta blockers, calcium-channel antagonists and digoxin, are poorly tolerated due to the preexistence of conduction impairment and the need for a faster heart rate to maintain an adequate cardiac output [62]. Their use has been linked to hypotension and HF exacerbation, particularly in more advanced stages of the disease [63,64]. Furthermore, calcium-channel antagonists and digoxin have been shown to bind to amyloid fibrils, leading to their accumulation and the subsequent potentiation of their activity, resulting in pro-arrhythmic effects [65,66,67]. Therefore, the use of these medications in CA must be considered with caution [68,69]. Due to the difficulties in rate control, rhythm control could be a valid alterative for symptom relief in CA, as AV synchrony through sinus rhythms is essential to maintain adequate stroke volumes. Among the antiarrhythmic drugs, amiodarone is preferred and could be a valuable alternative for rate control, because of the absence of a negative chronotropic effect [52]. Other antiarrhythmic drugs, like propafenone and flecainide, are contraindicated due to the risk of an iatrogenic 1:1 atrial flutter and reentrant ventricular arrhythmias. Dofetilide and sotalol have been proposed as alternatives to amiodarone [45,70].

### 3.3. Electrical Cardioversion

Electrical cardioversion (EC) remains the most effective treatment for sinus rhythm restoration, reporting an 88% acute success rate and 49% long-term success rate [66], similar to the general population. Of note, a higher rate of brady- and tachyarrhythmia has been demonstrated post-cardioversion, as evidence of the more diffuse mechanical and electrical impairment in CA [71,72]. 

### 3.4. Catheter Ablation

Given the above-reported concerns in the medical management of AF, interventional alternatives have to be considered. Catheter ablation has been demonstrated to be superior to antiarrhythmic drugs in reducing recurrence and improving quality of life, being recommended by the current guidelines in symptomatic paroxysmal or persistent AF [73]. To date, the safety and efficacy of catheter ablation in CA has been demonstrated only in small, retrospective studies [74,75]. Critical concerns include the increased risk of atrial thrombosis and hemodynamic imbalance due to the large volumes of fluid used during the procedure [76]. Therefore, better outcomes in terms of quality of life are achieved when ablation is performed in the early stages of the disease—more precisely, in the I-II NYHA class [77,78]. Procedures performed in advanced disease stages were also associated with an increased risk of complications. A recent study conducted by Ullah et al. [79] on 293 CA patients reported more adverse events, like pericardial effusion, and all-cause mortality with the same stroke rate during index admission for catheter ablation and at up to 30 days of follow-up compared to HF patients without CA. A significant recurrence rate after ablation has been reported, with a percentage of arrythmia-free patients of 40% at 1 year and up to 60% at 3 years [74,77]. When AF becomes permanent, AV nodal ablation remains the only alternative to avoid medications’ side effects, providing rate regularization and a subsequent improvement in cardiac output and quality of life [74,78]. In a study, the ventricular and atrial strain were used in addition to the LVEF to identify patients that may benefit from early AV ablation to avoid the development of tachycardia-induced cardiomyopathy [74].

### 3.5. Thromboembolic Risk Assessment

CA is intrinsically related to a higher thromboembolic risk compared to the general AF population [80,81]. The high prevalence of thromboembolic events such as stroke, transient ischemic attack and peripheral embolism is common in all CA subtypes [82,83,84], being, in such cases, the first presentation of the disease. wtATTR has been demonstrated to favor a more thrombogenic setting than vATTR [85], except for the V122l variant of vATTR [86]. On the other hand, AL has demonstrated an increased risk of intracardiac thrombi and thromboembolic events compared to ATTR [87]. The underlying pathophysiological mechanism is not completely understood, since intracardiac thrombi and thromboembolic events have been demonstrated in CA patients even during sinus rhythms [87]. Atrial dilatation and contraction impairment, as well as ventricular dysfunction, are responsible for the intracavitary turbulence that predisposes patients to clot formation regardless of the heart rhythm. Furthermore, atrial electromechanical dissociation has been demonstrated in patients with sinus rhythms [10]. An important contributing factor to the thromboembolic risk is systemic inflammation caused by the release of different pro-inflammatory cytokines and prothrombotic factors such as IL-6 and IL-8, which are produced in large quantities by fibroblasts under TTR stimulation and are involved in neo-angiogenesis, cell growth, apoptosis and survival [88].

The higher rate of thromboembolic events in AL compared to ATTR could be partially explained by the intrinsically more inflammatory environment and hypercoagulability that characterize multiple myeloma, as well as the side effects of the chemotherapy used for its treatment [89,90].

Anticoagulation therapy is therefore an essential part of CA management. Vilches et al. [80] demonstrated a reduction in thromboembolic events in ATTR patients with oral anticoagulation (OAC), advising against the use of the CHAD2DS2-VASc score for risk stratification because of its low predictive power. Indeed, a score of 0–1 was demonstrated to underestimate the risk of embolic events, and its use in non-AF patients did not predict embolic events during follow-up. Recent guidelines recommend OAC in all AF patients regardless of the CHAD2DS2-VASc score (class of recommendation I, LOE B) [91]. On the other hand, no recommendations are provided for patients with sinus rhythms. The assessment of the thromboembolic risk in these patients appears challenging because common risk factors, including advanced age, hypertension, diabetes mellitus and HF [92], do not show sufficient predictive power in CA. In contrast, LV dysfunction was significantly associated with thromboembolism in a study [82]. The evaluation of left atrial dysfunction has been also proposed by Akintoye et al. [93], reporting that the left atrial strain could predict 75% of the thromboembolic events that occurred before AF presentation. The left atrial appendage emptying velocity could be also a valuable alternative [82]. The management of AL appears to be more complex, where multiple factors, including nephrotic syndrome [83] and immunomodulatory drugs [94], contribute to increasing the thromboembolic risk.

**Figure 3:**
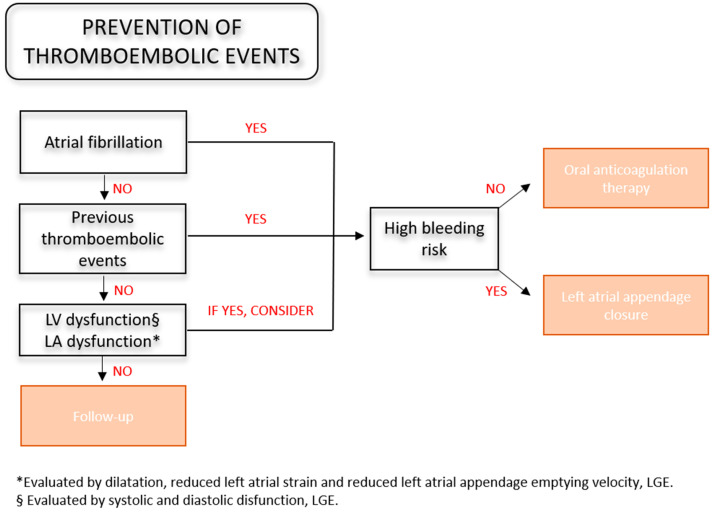
Decision tree for prevention of thromboembolic events in cardiac amyloidosis. LGE = late gadolinium enhancement; LA = left atrium; LV = left ventricle.

More extensive studies are needed to find alternatives to the CHAD2DS2-VASc score. Furthermore, a comprehensive assessment of the risk–benefit ratio of OAC in this population is necessary due to the concomitant higher bleeding risk. Indeed, the deposition of amyloid fibrils around vessels increases their fragility and the risk of rupture. Gastrointestinal involvement is not uncommon, and gastrointestinal bleeding with the need for blood transfusions is the most frequent hemorrhagic complication [95,96]. Interactions of disease-specific drugs with the coagulation pathway and anticoagulants contribute to increasing the hemorrhagic risk. Furthermore, liver and renal dysfunction, as well as falls due to dysautonomia, need to be considered [89]. However, no clear contraindication to OAC therapy has been identified, since fatal bleeding in anticoagulated CA patients has not been demonstrated and no correlation between bleeding events and worse outcomes has been established so far [97]. Regarding the choice of OAC, despite the higher INR lability of CA patients treated with VKA compared to the general population [87], no differences have been registered between vitamin K antagonists (VKA) and direct oral anticoagulants (DOACs) so far [98,99,100]. In CA patients, a transesophageal echocardiogram before cardioversion may be helpful in all cases, since left atrial appendage thrombosis and thromboembolic events despite adequate OAC therapy have been frequently reported in CA patients [71,81]. Figure 3 provides a decision-making algorithm for the prevention of thromboembolic events.

### 3.6. Left Atrial Appendage Closure

Studies on OAC’s effectiveness in patients with CA are limited and concerns regarding the concomitant higher thrombotic and bleeding risks compared to the general population need to be considered [98,100,101]. Gastrointestinal bleeding with the need for serial blood transfusion, coagulation disorders, cytopenia, hepatic and renal failure may contraindicate the use of OAC therapy [51]. Since >95% of the LA thrombi in AF patients are located in the left atrial appendage (LAA) [102], percutaneous catheter-based devices have been developed to exclude the LAA from the systemic circulation [103]. LAA closure (LAAC) is currently indicated in the case of a high bleeding risk or contraindications to OAC therapy [46]. The CAMYLAAC study [104] showed that LAAC in 40 ATTR patients did not differ regarding the incidence of ischemic/hemorrhagic stroke and major and minor bleeding compared to LAAC in non-CA patients. However, it can be speculated that left atrial cardiomyopathy associated with a restrictive physiology typical of advanced stages might increase the rate of thrombosis outside the LAA [105]. In the case of OAC failure, defined as cardioembolic events or LAA thrombosis despite adequate OAC therapy [106], the clinical guidelines do not provide clear recommendations, including a switch from one DOAC to another or intensifying the anticoagulation strategy [51]. Several studies have reported that the intensification of OAC to achieve thrombus resolution/effective secondary prevention is associated with a suboptimal result and a concomitant high bleeding incidence [107]. Early studies demonstrated that LAAC may be feasible and safe in this context [46], reporting optimistic results also in the long term [108,109]. However, LAAC is relatively contraindicated in the presence of LAA thrombosis, since these patients have been excluded in large studies [110]. Performing LAAC in the presence of LAA sludge or thrombosis may logically increase the risk of stroke and other complications, although this is not proven. Indeed, the most recent systematic review on LAAC in the presence of an LAA thrombus, including 16 studies and 58 patients, reported only one stroke and two device-related thromboses during a mean FU of 3.4 months [111]. Cerebral protection devices (CPDs) have been developed to mitigate the risk of cardioembolic embolism during transcatheter procedures [112]. They are mechanical barriers designed to cover the ostium of the supra-aortic branches in the aortic arch and descending aorta and allow surgeons to perform procedures that were previously contraindicated due to a high thromboembolic risk. Their use in tertiary care hospitals has demonstrated optimistic results, eliminating the risk of periprocedural thromboembolism during LAAC [113].

## 4. Ventricular Arrythmias

### 4.1. Etiopathogenesis and Epidemiology

Ventricular arrythmias (VA) are part of the natural history of CA, as their pathogenesis depends on different mechanisms, including the direct interaction of the amyloid fibrils with the conduction system, chronic inflammation, adverse remodeling and increased filling pressures [1,38]. Moreover, the heterogeneity of the viable myocardium in the ventricular walls due to the deposition of amyloid fibrils, interstitial fibrosis and myocardial ischemia [114] creates a substrate for re-entry circuits. Amyloid fibrils interact directly with the conduction system and cause vascular regional myocardial ischemia, promoting either functional or anatomical re-entry circuits. The autonomic dysregulation of the adrenergic system is ultimately involved. VAs are more frequently encountered in AL due to faster amyloid fibril deposition, which results in higher toxicity and a more irritating effect on the myocardium [48]. Moreover, disease-specific therapies like dexamethasone and chemotherapy are well known to be cardiotoxic with regard to VA occurrence [115].

The prevalence of VA in amyloidosis is higher than in the general population, with a 7.9-fold increased risk of VA and a 153-fold increased risk of new-onset ventricular tachycardia (VT) [22]. In a study, 72% of AL patients showed premature ventricular beats and up to 27% reported non-sustained VTs (nsVT) during 24 h Holter monitoring [116,117]. Accordingly, in a recent study, 34.97% of 143 AL patients had nsVT during Holter monitoring [118]. Less clear is the VA prevalence in ATTR because of the lack of data; in a cohort of 31 carriers (including both AL and ATTR) of implantable devices, the prevalence of nsVT and sustained VT was 74% and 19%, respectively [119]. Only recently, Knoll et al. reported, in 77 ATTRwt patients, the prevalence of 98% for premature ventricular beats and 44% for nsVT without sustained ventricular tachycardia [120].

Due to the small number of studies, the prognostic role of VA in CA is not clear and still a subject of discussion. Some studies have suggested that VA has no impact on overall survival and prognosis [121], probably due to imprecise patient selection [21], while others have reported a higher risk of SCD in the presence of ventricular couplets in ECG monitoring [122] and the need for appropriate ICD therapies [119]. In a study conducted by Dale et al. [123], the 1-year mortality rate in patients with CA and VT or ventricular fibrillation (VF) was 50%, with SCD accounting for 60% of deaths. In these studies, nsVT was positively correlated with an increased risk of SCD.

### 4.2. Pharmacological Therapy

Pharmacological therapy for VA in CA patients should be approached with caution, as previously reported. Thus far, the only treatments proven to reduce the arrhythmic burden are TTR stabilizers such as Tafamidis and Diflusinal [120,124]. Alternatively, catheter ablation has been performed in some case reports. Mlcochova et al. [125] described the successful ablation of drug-resistant VF in two patients, while Chung et al. [126] performed epicardial ablation in a patient with right ventricular amyloidosis. Notably, the electroanatomical mapping of the right ventricle has shown a high correlation between low-voltage electrograms (in both bipolar and unipolar configurations) and the extent of amyloid and fibrous tissue substitution observed in endomyocardial biopsy [127]. In specific cases, the preprocedural use of cardiac magnetic resonance imaging (MRI) to identify myocardial scars as the possible site of origin of VAs may be a valuable option [128]. However, further studies are needed to determine the real cost/benefit ratio of such procedures in these patients, considering their intrinsically high risk of hemodynamic destabilization, arrhythmia recurrence and poor prognosis.

### 4.3. Prevention of Sudden Cardiac Death

SCD is the most frequent cause of death in CA, accounting for up to two thirds of cases, particularly due to bradyarrhythmia [24,116]. In the past, skepticism was encountered regarding the primary implantation of implantable cardioverter defibrillators (ICDs), mainly due to the limited life expectancy associated with the disease and the high defibrillation threshold that may interfere with ICD functioning. However, in recent decades, the survival of those with CA has sharply increased [4] and the efficacy of ICDs has been proven [24,119,129], with appropriate and effective ICD therapies reported in up to a quarter of all implanted patients [37]. The 2019 HRS expert consensus [45] recommends secondary prevention implantation in patients who have survived a cardiac arrest and have a life expectancy greater than one year, while the 2022 ESC guidelines [130] recommend ICDs in patients with hemodynamically intolerable VTs after the careful evaluation of the advantages and benefits. Primary prevention ICD implantation is suggested by the 2019 HRS consensus [45] in AL with nsVT and by the Stanford Amyloid Center in patients with NYHA class I–III and a life expectancy > 1 year with a history of non-posturally mediated syncope and nsVT. The primary prevention of SCD in CA remains a matter of debate, and several studies discourage ICD implantation because clear evidence of its beneficial effect on survival has not yet been demonstrated [119,121,131].

**Figure 4:**
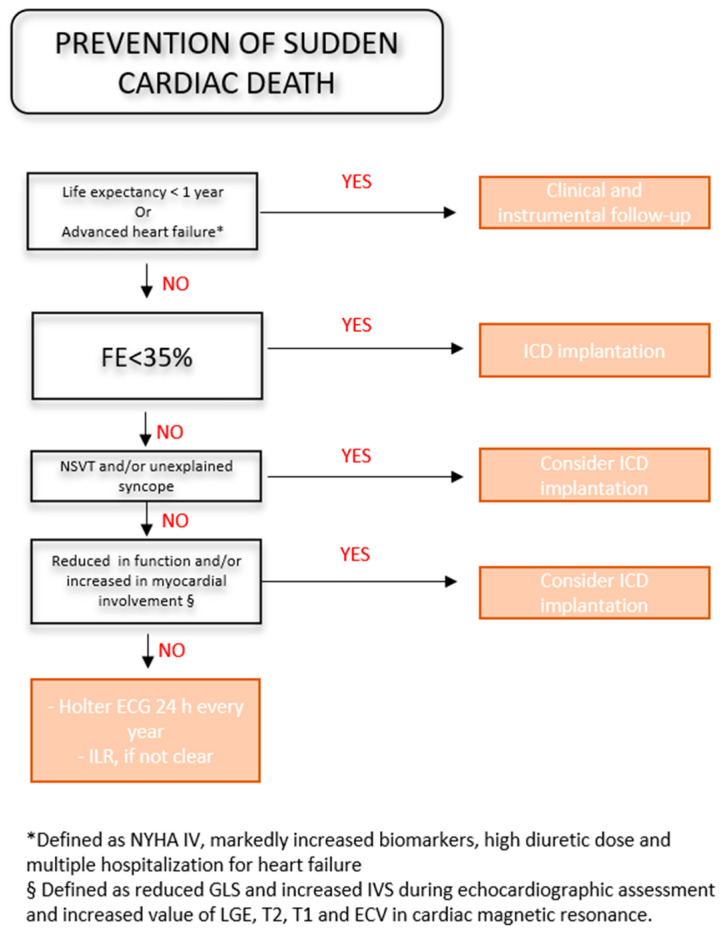
Decision tree for prevention of sudden cardiac death in cardiac amyloidosis. ECV = extracellular volume; EF = ejection fraction; GLS = global longitudinal strain; ICD = implantable cardioverter; ILR = implantable loop recorder; IVS = intraventricular septum thickness; LGE = late gadolinium enhancement; NSVT = non-sustained ventricular arrhythmia.

The lack of universally accepted criteria to identify patients that may benefit from preventive ICD implantation could be explained by incorrect patient selection, including those with end-stage HF [21]. Disease-specific criteria are needed to improve ICDs’ efficacy, such as for hypertrophic, arrhythmogenic or genetic cardiomyopathies [130]. Since CA’s prognosis is worse than that of other types of HF [4], the identification of those in which the SCD risk outweighs the risk of death for refractory HF is a concern. Biochemical samples such as cardiac troponin T, NT-proBNP, serum immunoglobulin free light chain and creatine clearance have been predictive of overall mortality [21,132,133], and, along with severe LV dysfunction, they are indicative of an advanced stage of HF and worse long-term outcomes [134], although not necessarily of an increased risk of SCD [135]. Several studies have demonstrated that VAs such as isolated ventricular beats or nsVT have been correlated with reduced survival in CA [117,122] and appropriate ICD shocks [136] also among CA patients [118]. Since AL is intrinsically characterized by a higher arrhythmic risk, the indication for ICD has to be weighted and compared to other CA types. Indeed, the HRS consensus considers the nsVT history as a strong risk criterion for SCD in AL. On the contrary, unlike other structural cardiomyopathies, in CA, unexplained syncope could be due to a number of different causes, like autonomic dysfunction, the hypovolemic status, bradyarrhythmia or atrial arrythmia, and only in a small part due to VAs [42]. It has therefore been suggested that prophylactic ICD implantation could be reasonable in unexplained syncope associated with an EP study that yielded negative results for conduction defects [37,39] and sustained atrial arrythmia [119]. However, non-inducibility in EP studies showed only minor prognostic value and has currently no role in CA patients, and monomorphic, sustained VTs have been rarely reported in CA [37]. Hamon et al. [121] demonstrated that LV GLS ≥ −15% predicted VA occurrence (*p* = 0.08), in contrast to the LVEF and left ventricular end diastolic diameter. Moreover, LV hypertrophy, a sign of amyloid infiltration, exhibited a correlation with the occurrence of VAs [116,137]. Tissue characterization by late gadolinium enhancement (LGE) has been negatively related to patients’ prognosis, while increases in T1 and T2 mapping remain the only diagnostic features without a predictive role [138]. A recent study reported a correlation between the T1 epicardial signal amplitude and extracellular volume and epicardial fractionated electrograms and prolonged repolarization [48]. In Figure 4, we provide a flowchart for ICD prophylactic implantation in CA.

### 4.4. Resynchronization Therapy

Cardiac resynchronization therapy (CRT) in amyloidosis is rarely proposed due to the supposed low probability of reverse remodeling caused by the infiltrative etiology. However, since the majority of patients will develop an indication to pacing during the follow-up, it may be useful to avoid PM-induced LV dysfunction. As reported in several studies, generally, right ventricular (RV) pacing > 20–40% has been associated with adverse outcomes, including incident AF, HF and increased mortality due to a pacing-induced cardiomyopathy [139,140]. Intraventricular dyssynchrony is detrimental in infiltrative CA, where the stroke volume is reduced and the cardiac output is rate-dependent. Donnellan et al. [129] demonstrated that, in ATTR patients, an RV paging burden > 40% was associated with worsening HF, the NYHA functional class, the mitral regurgitation severity and the LVEF, while biventricular (BiV) stimulation led to an improvement in these variables; similar results have been reported for AL amyloidosis [49]. Due to the relentless progression of the disease, it has been demonstrated that, at around 5 years from implantation, most of the patients become PM-dependent [43]. Therefore, early BiV stimulation might be a valuable solution. Although BiV is safe and effective in CA [49], it has been demonstrated that the CRT response in CA patients is inferior to that in in dilated cardiomyopathy patients (36% vs. 70%). Moreover, most of the patients do not meet the criteria for CRT because of the narrow QRS and preserved LVEF. Moreover, LV epicardial stimulation has been demonstrated to be pro-arrhythmic in non-responders [141], because of an increase in the action potential duration and transmural dispersion that determine the prolongation of the QTc and JT intervals. In this context, novel conduction system pacing (CSP) techniques encompassing the His bundle (HBP) and left bundle branch area pacing (LBBAP) may be valuable alternatives [142], with the significant narrowing of the QRS without the worsening of EF and NT-proBNP, as recently demonstrated in a cohort of 23 patients [143]. Since data on CSP in infiltrative diseases are limited [144], further studies are needed to explore its feasibility and long-term effects.

## 5. Limitations and Future Perspectives

CA has been considered an incidental finding with a poor or very poor prognosis for a long time. However, in the last few years, novelties in diagnosis, management and treatment have resulted in an improved survival rate. The prevention of SCD, for the development of either brady- or tachyarrhythmias, is a matter of debate and needs to be explored in larger, prospective studies. Indeed, as a predictive model for prophylactic PM implantation has been already proposed [40], only generic indications have been given for primary ICD implantation. A comprehensive analysis of clinical data, echocardiography, CMR and ECG monitoring is deemed necessary to quantify the arrhythmic risk of these patients. High expectations are indicated in CMR studies, including the correlation between the type/degree of myocardial impairment and the risk of SCD. The increased number of implantable devices associated with a prolonged life expectancy will lead to the increased use of BiV pacing and CSP, although more studies are needed to clarify their indications and to avoid overtreatment. The hemorrhagic and thromboembolic risks usually coexist in CA patients, and cerebrovascular events are frequently the first manifestations of CA, independently of the occurrence of AF. The consideration of initiating OAC therapy, even in sinus rhythms, for selected high-risk patients may be hypothesized and needs further investigation. On the other hand, the continuously expanding indications for LAAC may indicate that it is a valuable alternative in cases of OAC failure or OAC contraindication. Table 2 reports the major studies that have explored arrhythmia in cardiac amyloidosis.

## 6. Conclusions

The arrhythmic impact of CA has been widely underestimated in the past, but novel diagnostic methods and therapies have directed the spotlight towards this disease. In addition to HF management, arrythmias have been recognized as a critical part of the disease’s natural history. Both medical and interventional therapies have been studied and applied in real life, but, so far, the available data are not sufficient to provide universal guidelines.

## Figures and Tables

**Table 2:** Major studies about cardiac amyloidosis and arrhythmia.

Arrythmia	Study	Type of Study	Patients	Major Findings
Conduction system defects	Donnellan et al., 2020 [34]	Retrospective study	ATTRwt (261); ATTRv (108)	Incidence and prevalence of AV block
	Boldrini et al., 2013 [35]	Prospective observational study	AL (344)	Prevalence and prognostic implications of conduction delays
	Reisinger et al., 1997 [37]	Prospective observational study	AL (25)	Electrophysiologic abnormalities
	Algalarrondo et al., 2012 [30]	Retrospective study	ATTRv (262)	Prophylactic pacemaker implantation
	Barbhaiya et al., 2016 [39]	Prospective observational study	18	Intracardiac conduction, atrial arrythmias and ablation outcomes
	Porcari et al., 2022 [18]	Retrospective study	AL (127); ATTRv (66); ATTRwt (266)	Incidence and risk factors for pacemaker implantation
	Saturi et al., 2023 [40]	Retrospective study	AL (216); ATTR (571)	Prevalence, incidence and prognostic implications of pacemaker implantation
Atrial fibrillation	Longhi et al., 2015 [19]	Retrospective study	AL (123); ATTRv (94); ATTRwt (45)	Prevalence, incidence and prognostic implications of atrial fibrillation
	Papathamasiou et al., 2022 [50]	Retrospective study	AL (71); ATTRv (8); ATTRwt (54)	Prevalence and predictors of atrial fibrillation
	Donnellan et al., 2020 [20]	Retrospective study	ATTR (382)	Prevalence and incidence of atrial fibrillation
	Sanchis et al., 2019 [53]	Retrospective study	AL (115); ATTR (123)	Prevalence, incidence and prognostic implications of atrial fibrillation
	Rőcken et al., 2002 [55]	Prospective observational study	245	Role of isolated atrial amyloidosis in atrial fibrillation occurrence
	El-Am et al., 2019 [71]	Retrospective study	58	Outcomes of electrical cardioversion
	Black-Meier et al., 2020 [74]	Retrospective study	13	Feasibility of atrial fibrillation catheter ablation
	Amat-Santos et al., 2023 [104]	Retrospective study	ATTR (40)	Feasibility of left atrial appendage closure
Ventricular arrhythmias	Chen et al., 2022 [22]	Retrospective study	1130	Prevalence, incidence and prognostic implications of ventricular arrhythmias
	Palladini et al., 2001 [117]	Prospective observational study	AL (51)	Ventricular couplets as predictors of sudden death
	Varr et al., 2014 [119]	Retrospective study	31	Incidence of ventricular arrhythmias and role of ICD implantation
	Mlcochova et al., 2006 [125]	Case report	2	Catheter ablation of ventricular fibrillation
	Hamon et al., 2016 [121]	Prospective observational study	AL (12); ATTRv (27); ATTRwt (6)	Appropriate ICD therapy and survival predictors
	Kim et al., 2020 [131]	Retrospective study	AL (49); ATTR (41); Other (1)	Survival with and without a primary prevention ICD
	Lin et al., 2013 [21]	Retrospective study	AL (33); ATTRv (9); ATTRwt (10); Other (1)	Benefits of ICD therapy
	Donnellan et al., 2019 [129]	Retrospective study	ATTR (78)	Outcomes of implantable devices

## Data Availability

Not applicable.
